# Supplementary material for: Exploring the intersections of sexual stigma, poverty and mental health in HIV-negative gay, bisexual and other men who have sex with men in the United States
Source: PLOS Ment Health. 2024 Dec 30;1(7):e0000212. doi: 10.1371/journal.pmen.0000212 (PMC12798564; doi:10.1371/journal.pmen.0000212)
Supplement: S1 Text — (DOCX) [file pmen.0000212.s001.docx]

**S1 Appendix**

1. *Assessment of Measurement Invariance Across Survey Waves*

To ensure sexual stigma measurement consistency across survey waves, we assessed latent class measurement invariance [1]. Initially, we assessed whether the best fitting number of latent classes identified remained constant across survey years by fitting iterative LCA models with increasing numbers of latent lasses in data restricted to each survey cycle. Next, we assessed invariance in class-specific conditional response probabilities by comparing model fit (using AIC, BIC and SABIC) in the unconstrained model (i.e., model where the conditional response probabilities were allowed to vary freely across survey year) and fit in the partially constrained model (i.e., model where the response probabilities were fixed to be equal across survey years). The comparison involved examining estimated information criteria for both models, and a significant degradation in fit in the constrained model would suggest a lack of item-response invariance across survey years. Lastly, invariance in class prevalence was examined by comparing the fit indices estimated in the partially constrained model and the fully constrained model (i.e., model where both item-response probabilities and class prevalence were constrained to be equal across survey years). A poorer fit in the fully constrained model would indicate a lack of invariance in class prevalence rates across survey years.

1. *Quantification of Interaction on Additive & Multiplicative Scales*

The interaction between sexual stigma and poverty was quantified on both multiplicative and additive scales[2,3]. Multiplicative interaction assesses whether the outcome for groups with both factors present is greater or lesser than what would be expected based on the individual outcomes when each exposure exists separately. In contrast, additive interaction evaluates whether the presence of both factors results in an excess outcome compared to the sum of the individual outcomes when each exposure exists alone. Multiplicative interaction was calculated as PR11/ (PR10 * PR01), and additive interaction as PR11 - (PR10 + PR01 - 1) [3]. PR11 is the prevalence ratio (PR) comparing outcome prevalence in income-poor MSM with specific stigma experience to the prevalence in non-income-poor MSM with minimal sexual stigma experience (the reference group). PR01 compares outcome prevalence in income-poor MSM with minimal stigma experience to the reference group, and PR10 compares outcome prevalence in non-income-poor MSM with specific stigma experience to the reference group. Confidence intervals (95%) for these estimates were derived by bootstrapping (1000 samples used) [3].

1. *Record-level Probabilistic Bias Analysis & Adjustment*

Since sexual stigma patterns were assigned and not observed or known with certainty, there was a potential for misclassification. We assessed for and adjusted for potential bias due to stigma misclassification by implementing a record-level probabilistic bias adjustment technique to reconstruct the analytic sample and adjust for bias [4,5]. We operated under the assumption that misclassification in sexual stigma assignment was non-differential with respect to each study outcome and leveraged classification uncertainty rates, specifically probabilities of true latent class membership given the assigned class membership computed during sexual stigma measurement, to determine bias parameters [6]. Two sets of bias parameters were allocated: positive predictive value (PPV) of class assignment, assumed to follow a beta distribution, and reclassification probabilities, assumed to follow a Dirichlet distribution. To execute a single data reconstruction, we randomly selected bias parameters for each sexual stigma class based on the respective distribution functions. Subsequently, for each participant in the study, a Bernoulli trial was conducted using randomly selected class assignment PPV as the probability of correct assignment to determine whether everyone was correctly classified or not. Incorrectly assigned participants were reassigned using randomly sampled reclassification probabilities, establishing new class assignments for affected individuals. Outcome models, comprising individual factor impact models and the joint factors impact model, were then fitted in the fully reconstructed data. A total of 10,000 data reconstructions were conducted, each with a separate set of randomly sampled bias parameters. Estimates derived from fitting the outcome models in reconstructed data samples were aggregated and summarized using medians, which provided a reflection of the bias-adjusted point estimate measure of association, and 95% simulation intervals which systematic error and error due to uncertainty in bias parameters into account, ensuring comprehensive estimation of bias-adjusted outcomes. Bias-adjusted estimates with confidence intervals reflecting both random (data sampling error) and systematic errors were derived by fitting the regression models in bootstrapped data resampled with replacement from the reconstructed data.

References

1. Finch H. A Comparison of Statistics for Assessing Model Invariance in Latent Class Analysis. Open J Stat [Internet]. 2015 [cited 2023 Nov 27];5:191–210. Available from: http://www.scirp.org/journal/ojshttp://dx.doi.org/10.4236/ojs.2015.53022http://dx.doi.org/10.4236/ojs.2015.53022http://creativecommons.org/licenses/by/4.0/

2. VanderWeele T. Explanation in Causal Inference. 2015.

3. Vanderweele TJ, Knol MJ. A Tutorial on Interaction. Epidemiol Methods. 2014;3:33–72.

4. Lash TL, Schmidt M, Jensen AØ, Engebjerg MC. Methods to apply probabilistic bias analysis to summary estimates of association. Pharmacoepidemiol Drug Saf [Internet]. 2010 [cited 2023 Sep 13];19:638–44. Available from: https://pubmed.ncbi.nlm.nih.gov/20535760/

5. Lash TL, Fink AK. Semi-automated sensitivity analysis to assess systematic errors in observational data. Epidemiology [Internet]. 2003 [cited 2023 Sep 13];14:451–8. Available from: https://journals.lww.com/epidem/fulltext/2003/07000/semi_automated_sensitivity_analysis_to_assess.14.aspx

6. Asparouhov T, Muthén B. Auxiliary Variables in Mixture Modeling: A 3-Step Approach Using Mplus. Mplus Web Notes: No 15. 2013;
