# Supplementary material for: Exploring the intersections of sexual stigma, poverty and mental health in HIV-negative gay, bisexual and other men who have sex with men in the United States
Source: PLOS Ment Health. 2024 Dec 30;1(7):e0000212. doi: 10.1371/journal.pmen.0000212 (PMC12798564; doi:10.1371/journal.pmen.0000212)

**Exploring the Intersections of Sexual Stigma, Poverty and Mental Health in HIV-Negative Gay, Bisexual and Other Men Who Have Sex With Men in the United States**

**Authors:**

Udodirim N. Onwubiko, MBBS MPH^1^, Sarah M. Murray, PhD^2^, Amrita Rao, PhD^3^, Allison T. Chamberlain, PhD MS^1^, Travis H. Sanchez, PhD^1^, David Benkeser, PhD^4^, David P. Holland, MD MHS^5,6^, Samuel M. Jenness, PhD^1^, Stefan D. Baral, MD^3^

**Affiliations:**

^1^ Department of Epidemiology, Rollins School of Public Health, Emory University, Atlanta Georgia, United States

^2^ Department of Mental Health, Johns Hopkins Bloomberg School of Public Health, Baltimore, Maryland, United States

^3^ Center for Public Health and Human Rights, Department of Epidemiology, Johns Hopkins University, Baltimore, Maryland, United States

^4^ Department of Biostatistics and Bioinformatics, Rollins School of Public Health, Emory University, Atlanta Georgia, United States

^5^ Fulton County Board of Health, Atlanta Georgia, United States

^6^ Division of Infectious Disease, School of Medicine, Emory University, Atlanta Georgia, United States

**Supplemental Materials**

**Contents**

[Table A. American Men’s Internet Survey Items Used as Indicators of Individual-level Sexual Behavior Stigma Experiences (2018 – 2019) 3](#_Toc184719752)

[Table B. Endorsement of Sexual Stigma Experiences by Participant Age & Race, AMIS 2018-2019 4](#_Toc184719753)

[Table C. Latent Class Analysis of Sexual Stigma Experiences Among US MSM: Model Selection Criteria (Information Criteria, Likelihood Ratio Tests and Entropy), Conditional Response Probabilities and Class Prevalence (AMIS 2018 -2019) 5](#_Toc184719754)

[Table D. Assessment of Measurement Invariance of Sexual Behavior Stigma Across Survey Cohorts of Gay, Bisexual and Other Men who Have Sex with Men in the United States (AMIS 2018-2019) 6](#_Toc184719755)

[Table E. Within strata and systematic bias-adjusted estimates of associations between sexual stigma, income-poverty and mental health outcomes among gay, bisexual, and other men who have sex with men in the United States, AMIS 2018-2019 7](#_Toc184719756)

[Table F. Sensitivity Analysis: Latent Stigma Class Measurement using Recent versus None/Remote Sexual Stigma Experiences (AMIS 2018-2019) 8](#_Toc184719757)

[Table G. Sensitivity Analysis: Impacts of Income-to-Need Ratio Cutoff on Estimated Sexual Stigma Associations with Mental Health Incomes (AMIS 2018-2019) 9](#_Toc184719758)

[Figure A. Directed Acyclic Graph showing assumed relationships between dependent and independent variables used in assessing associations between sexual stigma patterns and mental health disorders among HIV-negative Men who have Sex with Men, AMIS 2019-2019. 10](#_Toc184719759)

[Figure B. Adjusted Prevalence Ratios of Sexual Stigma Association with Mental Health Outcomes Among US MSM and Variations by Income-Poverty, AMIS 2018-2019. Error bars represent 95% confidence interval; BA-Systematic Error = misclassification bias-adjusted estimates of association incorporating only uncertainty in bias parameters (systematic error); BA-Total Error = misclassification bias adjusted association estimates incorporating both systematic error and random error; Multiple Imputation = pooled estimates of association in 100 imputed datasets; SPD = Serious psychological distress. 11](#_Toc184719760)

# Table A. American Men’s Internet Survey Items Used as Indicators of Individual-level Sexual Behavior Stigma Experiences (2018 – 2019)

| **Survey Item** | **Stigma Construct** | **Type** | **Setting** |
| --- | --- | --- | --- |
| Have you ever felt afraid to go to health care services because you worry someone may learn you have sex with men? | Anticipated |  | Healthcare |
| Have you ever avoided going to health care services because you worry someone may learn you have sex with men? | Anticipated |  | Healthcare |
| Have you ever felt scared to be in public places because you have sex with men? | Anticipated |  | General/Social |
| Have you ever felt excluded from family activities because you have sex with men? | Perceived |  | Family |
| Have you ever felt that you were not treated well in a health center because someone knew that you have sex with men? | Perceived |  | Healthcare |
| Have you ever felt that the police refused to protect you because you have sex with men? | Perceived |  | Policing |
| Have you ever felt rejected by your friends because you have sex with men? | Perceived |  | Social |
| Have you ever been blackmailed by someone because they know that you have sex with men? | Enacted |  |  |
| Have you ever felt that family members have made discriminatory remarks or gossiped about you because you have sex with men? | Enacted | Verbal | Family |
| Have you ever heard health care providers gossiping about you (talking about you) because you have sex with men? | Enacted | Verbal | Healthcare |
| Have you ever been verbally harassed and felt it was because you have sex with men? | Enacted | Verbal | General/Social |
| Has someone ever physically hurt you (pushed, shoved, slapped, hit, kicked, choked, or otherwise physically hurt you)? | Enacted | Physical Assault | General/Social |
| Do you believe any of these experiences of physical violence was/were related to the fact that you have sex with men? | Enacted | Physical Assault | General/Social |
| Have you ever been forced to have sex when you did not want to? By forced, we mean physically forced, coerced to have sex, or penetrated with an object, when you did not want to? | Enacted | Sexual Assault | General/Social |
| Do you believe any of these experiences of sexual violence were related to the fact that you have sex with men? | Enacted | Sexual Assault | General/Social |

# Table B. Endorsement of Sexual Stigma Experiences by Participant Age & Race, AMIS 2018-2019

|  | **Age** | | | | | **Race/Ethnicity** | | | | |
| --- | --- | --- | --- | --- | --- | --- | --- | --- | --- | --- |
|  | **18-24** | **25-29** | **30-39** | **40 plus** | **p-value** | **Black, NH** | **Hispanic** | **Other/**  **Multiple** | **White, NH** | **p-value** |
|  | **n (%)** | **n (%)** | **n (%)** | **n (%)** |  | **n (%)** | **n (%)** | **n (%)** | **n (%)** |  |
| N | 3956 | 2418 | 2267 | 3859 |  | 1146 | 1854 | 972 | 8315 |  |
| **Endorsed Sexual Behavior Stigma Experience:**  **Have you ever ______ because you have sex with men?** |  |  |  |  |  |  |  |  |  |  |
| felt excluded from family activities (A1) | 1439 (39%) | 832 (37%) | 743 (35%) | 1098 (30%) | < 0.001 | 295 (28%) | 559 (33%) | 328 (37%) | 2860 (36%) | < 0.001 |
| felt that family members have made discriminatory remarks or gossiped about you (A2) | 2084 (58%) | 1195 (55%) | 1024 (50%) | 1522 (44%) | < 0.001 | 541 (52%) | 877 (54%) | 434 (50%) | 3881 (51%) | 0.216 |
| felt rejected by your friends (A3) | 1136 (30%) | 637 (27%) | 614 (28%) | 965 (27%) | 0.019 | 244 (22%) | 407 (23%) | 265 (28%) | 2364 (30%) | < 0.001 |
| felt afraid to go to health care services because you worry someone may learn (B1) | 1140 (29%) | 688 (29%) | 623 (28%) | 890 (23%) | < 0.001 | 286 (25%) | 482 (26%) | 251 (26%) | 2258 (27%) | 0.348 |
| avoided going to health care services because you worry someone may learn (B2) | 898 (23%) | 553 (23%) | 494 (22%) | 697 (18%) | < 0.001 | 219 (19%) | 409 (22%) | 197 (21%) | 1766 (21%) | 0.242 |
| heard health care providers gossiping about you (talking about you) (B3) | 128 (3%) | 107 (5%) | 120 (5%) | 168 (4%) | 0.001 | 46  (4%) | 75  (4%) | 43  (5%) | 344 (4%) | 0.956 |
| felt that you were not treated well in a health center because someone knew (B4) | 282 (7%) | 233 (10%) | 286 (13%) | 357 (10%) | < 0.001 | 62  (6%) | 151 (8%) | 74  (8%) | 844 (10%) | < 0.001 |
| felt that the police refused to protect you (C1) | 236 (6%) | 150 (7%) | 207 (10%) | 355 (10%) | < 0.001 | 83  (8%) | 151 (9%) | 80  (9%) | 604 (8%) | 0.341 |
| felt scared to be in public places (C2) | 1718 (44%) | 1027 (43%) | 886 (40%) | 1189 (31%) | < 0.001 | 308 (27%) | 709 (39%) | 398 (42%) | 3321 (41%) | < 0.001 |
| been verbally harassed and felt it was (C3) | 1938 (50%) | 1162 (49%) | 1054 (47%) | 1640 (43%) | < 0.001 | 370 (33%) | 790 (43%) | 427 (45%) | 4114 (50%) | < 0.001 |
| been blackmailed by someone (C4) | 652 (17%) | 352 (15%) | 256 (11%) | 406 (11%) | < 0.001 | 152 (13%) | 223 (12%) | 145 (15%) | 1112 (14%) | 0.196 |
| Has someone ever physically hurt you (pushed, shoved, slapped, hit, kicked, choked, or otherwise physically hurt you) (D1) | 628 (17%) | 367 (16%) | 380 (17%) | 691 (18%) | 0.034 | 139 (12%) | 291 (16%) | 163 (17%) | 1417 (18%) | < 0.001 |
| been forced to have sex when you did not want to (i.e., physically forced, coerced to have sex, or penetrated with an object, when you did not want to) (D3) | 484 (13%) | 246 (11%) | 208 (10%) | 375 (10%) | < 0.001 | 103 (9%) | 181 (11%) | 116 (13%) | 879 (11%) | 0.063 |

# Table C. Latent Class Analysis of Sexual Stigma Experiences Among US MSM: Model Selection Criteria (Information Criteria, Likelihood Ratio Tests and Entropy), Conditional Response Probabilities and Class Prevalence (AMIS 2018 -2019)

| **Model** | **AIC** | **BIC** | **SABIC** | **Entropy** | **VLMR**  **p-value** | **BLRT**  **p-value** |
| --- | --- | --- | --- | --- | --- | --- |
| 2-class | 135910.7 | 136111.4 | 136025.6 | 0.78 | 0 | 0 |
| 3-class | 131695.1 | 131999.9 | 131869.6 | 0.81 | 0 | 0 |
| 4-class | 128847.1 | 129255.9 | 129081.1 | 0.81 | 0 | 0 |
| 5-class | 127675.0 | 128187.9 | 127968.6 | 0.79 | 0 | 0 |
| 6-class | 126697.3 | 127314.2 | 127050.5 | 0.77 | 0 | 0 |
| 7-class | 125989.0 | 126710.0 | 126401.8 | 0.76 | 0 | 0 |
|  |  |  |  |  |  |  |
| **Conditional Response Probabilities (4-class LCA)** | **Class 1** | **Class 2** | **Class 3** | **Class 4** |  |  |
| felt excluded from family activities (A1) | 0.84 | 0.3 | 0.59 | 0.04 |  |  |
| felt that family members have made discriminatory remarks or gossiped about you (A2) | 0.94 | 0.48 | 0.8 | 0.17 |  |  |
| felt rejected by your friends (A3) | 0.75 | 0.28 | 0.38 | 0.06 |  |  |
| felt afraid to go to health care services because you worry someone may learn (B1) | 0.85 | 0.94 | 0.07 | 0.05 |  |  |
| avoided going to health care services because you worry someone may learn (B2) | 0.78 | 0.86 | 0.01 | 0.01 |  |  |
| heard health care providers gossiping about you (talking about you) (B3) | 0.24 | 0.03 | 0.03 | 0.01 |  |  |
| felt that you were not treated well in a health center because someone knew (B4) | 0.47 | 0.08 | 0.08 | 0.01 |  |  |
| felt that the police refused to protect you (C1) | 0.32 | 0.02 | 0.11 | 0.01 |  |  |
| felt scared to be in public places (C2) | 0.88 | 0.38 | 0.57 | 0.11 |  |  |
| been verbally harassed and felt it was (C3) | 0.96 | 0.34 | 0.73 | 0.16 |  |  |
| been blackmailed by someone (C4) | 0.39 | 0.15 | 0.16 | 0.04 |  |  |
| Has someone ever physically hurt you (pushed, shoved, slapped, hit, kicked, choked, or otherwise physically hurt you) (D1) | 0.56 | 0.05 | 0.28 | 0.02 |  |  |
| been forced to have sex when you did not want to (i.e., physically forced, coerced to have sex, or penetrated with an object, when you did not want to) (D3) | 0.38 | 0.07 | 0.15 | 0.02 |  |  |
| **Class Prevalence** | 11.9% | 13.3% | 33.6% | 41.2% |  |  |

**Abbreviations**: AIC = Akaike information criterion; BIC = Bayesian information criterion; SABIC = sample-size-adjusted BIC; VLMR = Vuong-Lo-Mendell-Rubin test; BLRT = bootstrapped likelihood ratio test.

Latent stigma classes: Class 1: labeled “the Diverse Sexual Stigma Class” – Characterized by high probabilities of endorsing various types of stigma experiences across different settings. Class 2: labeled “the Anticipated Healthcare Predominant Sexual Stigma Class” – Defined by a particularly high probability of endorsing anticipatory stigma in healthcare settings. Class 3: labeled “the Family and General Social Sexual Stigma Class” – Marked by moderate-to-high probabilities of endorsing stigma related to family and general social settings, but not in healthcare settings. Class 4: labeled “the Minimal Sexual Stigma Class” – marked by generally low probabilities of endorsing any of the surveyed sexual stigma experiences

# Table D. Assessment of Measurement Invariance of Sexual Behavior Stigma Across Survey Cohorts of Gay, Bisexual and Other Men who Have Sex with Men in the United States (AMIS 2018-2019)

| **Model** | **AIC** | **BIC** | **SABIC** | **Entropy** | **VLMR**  **p-value** | **BLRT**  **p-value** |
| --- | --- | --- | --- | --- | --- | --- |
| **Assessment of Measurement Invariance in Number of Latent Classes Identified** |  |  |  |  |  |  |
| 1. **2018 Survey Year** |  |  |  |  |  |  |
| 2-class model | 64389.1 | 64570.0 | 64484.2 | 0.77 | 0 | 0 |
| 3-class model | 62385.4 | 62660.2 | 62529.9 | 0.81 | 0 | 0 |
| 4-class model | 61020.4 | 61389.0 | 61214.2 | 0.82 | 0 | 0 |
| 5-class model | 60411.5 | 60873.9 | 60654.7 | 0.79 | 0 | 0 |
| 6-class model | 59940.1 | 60496.4 | 60232.7 | 0.77 | 0 | 0 |
| 7-class model | 59670.4 | 60320.5 | 60012.3 | 0.76 | 0.0102 | 0 |
| 1. **2019 Survey Year** |  |  |  |  |  |  |
| 2-class model | 71494.7 | 71677.7 | 71591.9 | 0.78 | 0 | 0 |
| 3-class model | 69283.9 | 69561.7 | 69431.5 | 0.81 | 0 | 0 |
| 4-class model | 67818.0 | 68190.7 | 68015.9 | 0.81 | 0 | 0 |
| 5-class model | 67232.3 | 67699.8 | 67480.5 | 0.79 | 0 | 0 |
| 6-class model | 66769.6 | 67332.0 | 67068.2 | 0.77 | 0 | 0 |
| 7-class model | 66351.4 | 67008.7 | 66700.4 | 0.76 | 0.001 | 0 |
|  |  |  |  |  |  |  |
| **Assessment of Invariance in Conditional Response Probabilities and Class Sizes** |  |  |  |  |  |  |
| Model 1 | 146145.1 | 146970.2 | 146617.4 |  |  |  |
| Model 2 | 146154.0 | 146592.6 | 146405.1 |  |  |  |
| Model 3 | 146153.8 | 146570.1 | 146392.1 |  |  |  |

**Abbreviations:** AIC = Akaike information criterion; BIC = Bayesian information criterion; SABIC = sample-size-adjusted BIC; VLMR = Vuong-Lo-Mendell-Rubin test; BLRT = bootstrapped likelihood ratio test

Assessment of measurement invariance conducted in stages (see Appendix). Invariance in best fitting number of latent class assessed by determining whether the 4-class model remained best fitting in data restricted to either survey year. Invariance in conditional response probabilities assessed by comparing fit statistics in models 1 and 2 (**Model 1** = Unconstrained model where response probabilities and class prevalences were allowed to freely vary across survey years; **Model 2** = Partially constrained model where conditional response probabilities were constrained to be equal across survey years while class prevalences were allowed to vary). Invariance in class prevalence assessed by comparing fit statistics in models 2 and 3 (**Model 3** = Fully constrained model where both conditional response probabilities and class prevalences wee constrained to be equal across survey years). Lack of degradation in model fit (information criteria did not increase appreciably) indicated invariance in measurement across survey years.

# Table E. Within strata and systematic bias-adjusted estimates of associations between sexual stigma, income-poverty and mental health outcomes among gay, bisexual, and other men who have sex with men in the United States, AMIS 2018-2019

| **Income Poverty Strata** | **Sexual Behavior Stigma Class (SBSC)** | **Within Strata Estimates** | | **Common Reference Group** | | |
| --- | --- | --- | --- | --- | --- | --- |
|  |  |  |  | **Misc. Bias-Adjusted^a^**  **(Systematic Error only)** | **Misc. Bias-Adjusted**  **(Total error)^b^** | **Pooled Estimates**  **Multiple Imputation^c^** |
|  |  | **Crude PR (95% CI)** | **Adjusted PR (95% CI)** | **Adjusted PR (95% SI)** | **Adjusted PR (95% SI)** | **Adjusted PR (95% CI)** |
| **Outcome = Serious Psychological Distress (SPD)** | | | | | | |
| Poor | Diverse | 2.97 (2.36, 3.74) | 2.80 (2.21, 3.53) | 4.31 (3.95, 4.71) | 4.31 (3.60, 5.15) | 3.11 (2.60, 3.71) |
|  | AHP | 2.06 (1.59, 2.67) | 1.85 (1.42, 2.41) | 3.04 (2.72, 3.40) | 3.04 (2.42, 3.77) | 2.17 (1.76, 2.68) |
|  | FGSP | 2.05 (1.66, 2.52) | 1.96 (1.59, 2.42) | 3.06 (2.82, 3.33) | 3.06 (2.59, 3.64) | 2.31 (1.99, 2.69) |
|  | Minimal | *Ref* | *Ref* | 1.71 (1.56, 1.88) | 1.71 (1.39, 2.08) | 1.39 (1.16, 1.68) |
| Not Poor | Diverse | 3.99 (3.41, 4.66) | 3.89 (3.32, 4.55) | 3.44 (3.15, 3.79) | 3.44 (2.92, 4.08) | 2.69 (2.33, 3.10) |
|  | AHP | 2.09 (1.75, 2.50) | 1.99 (1.66, 2.38) | 1.95 (1.75, 2.18) | 1.95 (1.61, 2.37) | 1.56 (1.32, 1.85) |
|  | FGSP | 2.29 (1.99, 2.63) | 2.17 (1.88, 2.49) | 1.97 (1.79, 2.18) | 1.97 (1.68, 2.33) | 1.69 (1.49, 1.92) |
|  | Minimal | *Ref* | *Ref* | *Ref* | *Ref* | *Ref* |
| **Outcome = Suicidal Ideation in past year** | | | | | | |
| Poor | Diverse | 2.91 (2.25, 3.75) | 2.74 (2.11, 3.54) | 3.75 (3.42, 4.09) | 3.74 (3.08, 4.54) | 2.62 (2.14, 3.19) |
|  | AHP | 1.89 (1.41, 2.54) | 1.72 (1.28, 2.31) | 2.45 (2.16, 2.77) | 2.45 (1.90, 3.10) | 1.78 (1.41, 2.27) |
|  | FGSP | 2.01 (1.60, 2.53) | 1.92 (1.52, 2.42) | 2.69 (2.46, 2.94) | 2.69 (2.25, 3.21) | 2.00 (1.70, 2.35) |
|  | Minimal | *Ref* | *Ref* | 1.48 (1.35, 1.62) | 1.48 (1.18, 1.82) | 1.25 (1.02, 1.51) |
| Not Poor | Diverse | 3.58 (3.04, 4.22) | 3.50 (2.97, 4.13) | 3.22 (2.95, 3.51) | 3.22 (2.72, 3.80) | 2.42 (2.08, 2.83) |
|  | AHP | 2.03 (1.69, 2.44) | 1.96 (1.63, 2.35) | 1.88 (1.68, 2.09) | 1.88 (1.54, 2.28) | 1.53 (1.29, 1.81) |
|  | FGSP | 2.14 (1.86, 2.46) | 2.04 (1.77, 2.36) | 1.95 (1.76, 2.14) | 1.95 (1.66, 2.29) | 1.61 (1.41, 1.82) |
|  | Minimal | *Ref* | *Ref* | *Ref* | *Ref* | *Ref* |
| **Outcome = Suicide Attempt in past year** | | | | | | |
| Poor | Diverse | 4.75 (2.64, 8.52) | 4.57 (2.54, 8.25) | 10.74 (8.51, 13.57) | 10.82 (6.07, 20.12) | 5.73 (3.52, 9.32) |
|  | AHP | 1.93 (0.92, 4.04) | 1.73 (0.82, 3.63) | 4.35 (2.99, 6.28) | 4.34 (1.80, 9.20) | 2.49 (1.28, 4.84) |
|  | FGSP | 1.68 (0.92, 3.06) | 1.55 (0.85, 2.84) | 3.94 (3.10, 5.16) | 3.95 (2.16, 7.48) | 2.43 (1.48, 4.01) |
|  | Minimal | *Ref* | *Ref* | 2.40 (1.90, 2.98) | 2.39 (1.13, 4.77) | 1.66 (0.93, 2.95) |
| Not Poor | Diverse | 3.93 (2.23, 6.92) | 3.92 (2.19, 7.00) | 3.63 (2.76, 4.70) | 3.63 (1.85, 7.09) | 2.45 (1.42, 4.21) |
|  | AHP | 1.24 (0.58, 2.67) | 1.21 (0.56, 2.61) | 1.23 (0.82, 1.85) | 1.23 (0.44, 2.78) | 1.12 (0.57, 2.23) |
|  | FGSP | 2.47 (1.52, 4.03) | 2.34 (1.42, 3.85) | 2.30 (1.77, 3.08) | 2.32 (1.34, 4.35) | 1.69 (1.08, 2.63) |
|  | Minimal | *Ref* | *Ref* | *Ref* | *Ref* | *Ref* |

**Abbreviations**: CI = confidence interval; SI = simulation interval; PR = prevalence ratio; AHP = anticipated healthcare predominant; FGSP = family/general social predominant.

^a^Sexual stigma misclassification bias adjustment using record-level probabilistic bias analysis with 95% SI incorporating uncertainty in bias parameters only; ^b^Sexual stigma misclassification bias adjustment with 95% SI incorporating both uncertainty in bias parameters and random error; ^c^Multiple imputation to address potential selection bias due to missing data. Note: All adjusted models were adjusted for participant age, race/ethnicity, nativity, US region of residence and urbanicity

# Table F. Sensitivity Analysis: Latent Stigma Class Measurement using Recent versus None/Remote Sexual Stigma Experiences (AMIS 2018-2019)

| **Model** | **AIC** | **BIC** | **SABIC** | **Entropy** | **VLMR**  **p-value** | **BLRT**  **p-value** | **Minimum class size**  **n (%)** |
| --- | --- | --- | --- | --- | --- | --- | --- |
| 2-class | 70109.1 | 70309.8 | 70224.0 | 0.79 | 0 | 0 | 3004 (24) |
| 3-class | 67830.9 | 68135.7 | 68005.4 | 0.85 | 0 | 0 | 758 (6.1) |
| 4-class | 66671.6 | 67080.4 | 66905.6 | 0.85 | 0 | 0 | 347 (2.8) |
| 5-class | 65908.6 | 66421.5 | 66202.2 | 0.86 | 0 | 0 | 303 (2.4) |
| 6-class | 65562.8 | 66179.7 | 65916.0 | 0.83 | 0 | 0 | 246 (2) |
| 7-class | 65320.4 | 66041.4 | 65733.2 | 0.84 | 0 | 0 | 67 (0.5) |

**Abbreviations**: AIC = Akaike information criterion; BIC = Bayesian information criterion; SABIC = sample-size-adjusted BIC; VLMR = Vuong-Lo-Mendell-Rubin test; BLRT = bootstrapped likelihood ratio test

# Table G. Sensitivity Analysis: Impacts of Income-to-Need Ratio Cutoff on Estimated Sexual Stigma Associations with Mental Health Incomes (AMIS 2018-2019)

| **Income Poverty Strata** | **Sexual Behavior Stigma Class (SBSC)** | **Adjusted Prevalence Ratios (95% Confidence Intervals)** | | |
| --- | --- | --- | --- | --- |
|  |  | **INR cutoff=1** | **INR cutoff=2** | **INR cutoff=3** |
| **Outcome = Serious Psychological Distress (SPD)** | | | | |
| Poor | Diverse | 4.17 (3.34, 5.20) | 4.73 (3.89, 5.74) | 5.76 (4.76, 6.96) |
|  | AHP | 3.03 (2.35, 3.91) | 3.14 (2.49, 3.95) | 3.39 (2.75, 4.19) |
|  | FGSP | 3.15 (2.64, 3.76) | 3.32 (2.81, 3.92) | 3.77 (3.17, 4.48) |
|  | Minimal | 1.44 (1.13, 1.83) | 1.69 (1.37, 2.08) | 1.88 (1.55, 2.28) |
| Not Poor | Diverse | 3.79 (3.27, 4.39) | 3.89 (3.32, 4.55) | 4.27 (3.50, 5.20) |
|  | AHP | 1.92 (1.62, 2.27) | 1.99 (1.66, 2.38) | 2.15 (1.71, 2.70) |
|  | FGSP | 2.10 (1.85, 2.40) | 2.17 (1.88, 2.49) | 2.25 (1.88, 2.70) |
|  | Minimal | *Ref* | *Ref* | *Ref* |
| **Outcome = Suicidal Ideation in past year** | | | | |
| Poor | Diverse | 3.88 (3.05, 4.94) | 4.00 (3.24, 4.95) | 4.70 (3.86, 5.73) |
|  | AHP | 2.56 (1.92, 3.41) | 2.52 (1.95, 3.26) | 2.86 (2.29, 3.56) |
|  | FGSP | 2.56 (2.10, 3.12) | 2.81 (2.36, 3.36) | 3.10 (2.60, 3.70) |
|  | Minimal | 1.41 (1.09, 1.81) | 1.46 (1.17, 1.83) | 1.59 (1.31, 1.93) |
| Not Poor | Diverse | 3.45 (2.95, 4.03) | 3.50 (2.97, 4.13) | 3.66 (2.98, 4.49) |
|  | AHP | 1.91 (1.61, 2.28) | 1.96 (1.63, 2.35) | 1.99 (1.58, 2.50) |
|  | FGSP | 2.08 (1.81, 2.38) | 2.04 (1.77, 2.36) | 2.08 (1.74, 2.49) |
|  | Minimal | *Ref* | *Ref* | *Ref* |
| **Outcome = Suicide Attempt in past year** | | | | |
| Poor | Diverse | 11.83 (6.69, 20.92) | 11.30 (6.58, 19.41) | 13.28 (6.73, 26.21) |
|  | AHP | 3.97 (1.78, 8.89) | 4.26 (2.10, 8.66) | 4.43 (2.02, 9.68) |
|  | FGSP | 4.08 (2.29, 7.27) | 3.83 (2.19, 6.69) | 4.97 (2.53, 9.73) |
|  | Minimal | 2.92 (1.53, 5.56) | 2.47 (1.31, 4.65) | 3.21 (1.59, 6.47) |
| Not Poor | Diverse | 4.71 (2.79, 7.95) | 3.92 (2.19, 7.00) | 5.58 (2.53, 12.30) |
|  | AHP | 1.56 (0.80, 3.03) | 1.21 (0.56, 2.61) | 1.60 (0.56, 4.62) |
|  | FGSP | 2.38 (1.48, 3.83) | 2.34 (1.42, 3.85) | 3.35 (1.67, 6.74) |
|  | Minimal | *Ref* | *Ref* | *Ref* |

**Abbreviations**: CI = confidence interval; SI = simulation interval; PR = prevalence ratio; AHP = anticipated healthcare predominant; FGSP = family/general social predominant.

# Figure A. Directed Acyclic Graph showing assumed relationships between dependent and independent variables used in assessing associations between sexual stigma patterns and mental health disorders among HIV-negative Men who have Sex with Men, AMIS 2019-2019.

Sexual Stigma Class

Mental Health Disorders

Race/Ethnicity

Cultural Identity & Structural Racism Experience (U)

Income Poverty

**Stigma Indicators**

**(k=13)**

Age

*Stigma measurement model*

US Region

Urbanicity

Nativity

Homelessness

# Figure B. Adjusted Prevalence Ratios of Sexual Stigma Association with Mental Health Outcomes Among US MSM and Variations by Income-Poverty, AMIS 2018-2019. Error bars represent 95% confidence interval; BA-Systematic Error = misclassification bias-adjusted estimates of association incorporating only uncertainty in bias parameters (systematic error); BA-Total Error = misclassification bias adjusted association estimates incorporating both systematic error and random error; Multiple Imputation = pooled estimates of association in 100 imputed datasets; SPD = Serious psychological distress.


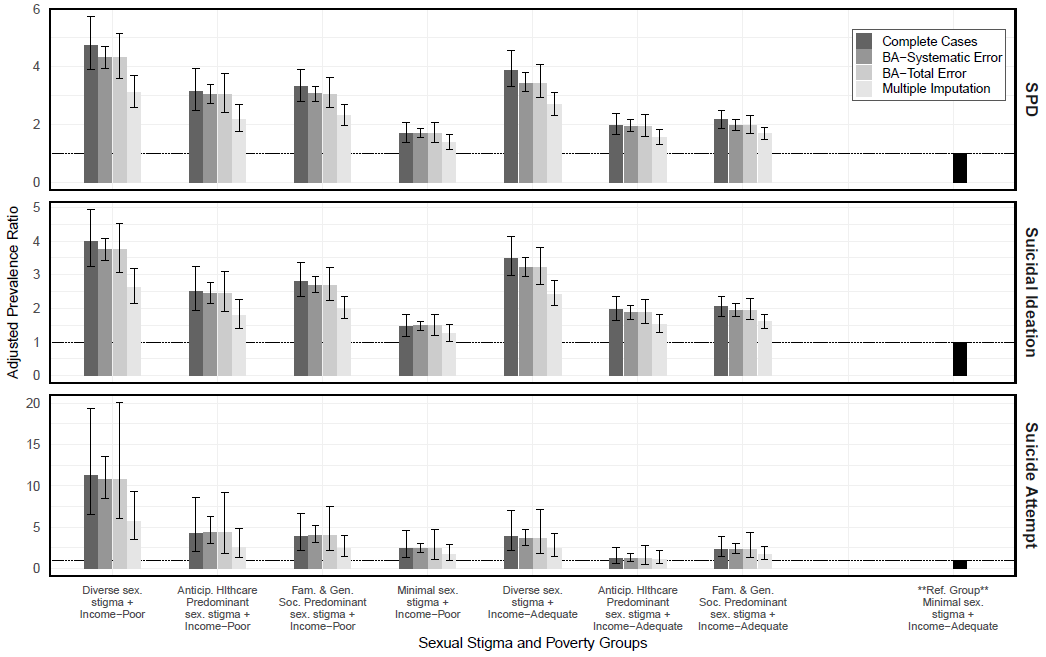

Supplement: S2 Text — (DOCX) [file pmen.0000212.s002.docx]
