# Supplementary material for: Exploring the intersections of sexual stigma, poverty and mental health in HIV-negative gay, bisexual and other men who have sex with men in the United States
Source: PLOS Ment Health. 2024 Dec 30;1(7):e0000212. doi: 10.1371/journal.pmen.0000212 (PMC12798564; doi:10.1371/journal.pmen.0000212)
Supplement: Attachment — Submitted filename: 02_Response to Reviewers.docx [file pmen.0000212.s003.docx]

**Reviewer 1**

Dear authors
I have reviewed the manuscript titled "Intersecting Realities: Understanding Stigma, Poverty, and Mental Health in HIV-Negative Men who have Sex with Men in the United States." The study explores the intersection of sexual stigma, poverty, and mental health among HIV-negative men who have sex with men (MSM) in the United States using data from the 2018 and 2019 American Men's Internet Survey (AMIS).
Without a doubt, this study addresses highly relevant social and public health issues, namely sexual stigma, poverty, and mental health in men who have sex with men (MSM). These factors are critical because MSM historically face high levels of discrimination and stigmatization, contributing to significant disparities in mental health and access to healthcare services.

However, I have some concerns, which I will comment on below:

1. **Comment:** The manuscript provides a strong rationale for the study. However, it would be beneficial to focus more on the specific gap that this study addresses within the current body of literature, especially regarding HIV-negative MSM. For instance, the authors could discuss existing studies' limitations to underscore this work's significance.

**Response:** Thank you for your feedback. We agree that further emphasizing the specific gap our study addresses is essential. The primary gap we aim to fill is the limited research on how sexual stigma and socioeconomic disadvantage interact to affect mental health outcomes among sexual minorities, particularly HIV-negative MSM. While prior studies have examined these factors separately, few have explored their combined impact on mental health disparities. To better clarify our contribution, we have revised the background section to more explicitly outline these gaps and the limitations of existing studies, underscoring the significance of this work. Specifically, we address how this study aims to advance understanding of the synergistic relationship between stigma and socioeconomic status, offering new insights into mental health disparities that can guide more effective interventions. We included the following paragraph in the manuscript background section:

*“Several studies have examined how stigma and poverty influence mental health outcomes.[31–34] While much research has highlighted sexual stigma as a major factor driving higher rates of depression and other mental health issues among sexual minorities [31,33,35] others have investigated socioeconomic disadvantage (e.g., education, income, occupation), consistently linking lower socioeconomic status to poorer mental health.[31,32] However, evidence indicates that sexual stigma and economic hardship are interrelated phenomena. Stigma can drive economic challenges through structural pathways that limit the resources available to stigmatized individuals, while poverty may amplify the negative effects of stigma by further restricting access to vital support resources.[27,28,30]. Despite this intersection, few studies have explored the combined impact of sexual stigma and poverty on the mental health of HIV-negative US GBM, a group that may lack the enhanced mental health services available to those living with HIV.[36].”[Manuscript clean copy, Background section, line 44-55]*

2. **Comment:** Regarding the analytical strategy, while I appreciate the use of latent class analysis (LCA) to capture heterogeneity in the experiences of sexual stigma among MSM, I have some reservations. My main concern is about the use of a combined dataset, as I believe it might be impossible to analyze the overlap between the 2018 and 2019 datasets. This problem raises the question of to what extent this overlap might bias the study's results. Specifically, there is a risk of overrepresenting certain groups, which could, in turn, skew the analysis of the relationship between the latent classes and other variables. Given the substantial sample sizes of both datasets, combining them seems unnecessary and wrong. I suggest using only one dataset or separately presenting the results for both.

**Response:** We sincerely appreciate the reviewer’s thoughtful feedback regarding the use of a combined dataset and would like to offer further clarification on our approach. The reviewer raised concerns about potential overlap between the 2018 and 2019 datasets, and we seek to clarify what specific overlap might be at issue. If the concern is overlapping data collection timelines, we assure the reviewer that this is not relevant in our case. As outlined in the methods section, the AMIS data collection cycles were distinct: data for 2018 were collected from September to December, while data for 2019 were collected from August to December. There was no overlap in the actual data collection periods. If the concern is about repeat respondents completing the surveys in both years, existing research using combined AMIS datasets suggests that this is minimal. For instance, Godreau et al. (2021), who combined AMIS data from 2017 and 2018, found that only about 10% of the 2018 responses were from individuals who had completed the survey the prior year (Godreau et al., 2021 <https://www.ncbi.nlm.nih.gov/pmc/articles/PMC8528882/>). This suggests that the likelihood of repeat participation is low and unlikely to introduce significant bias.

Another concern raised was the potential for overrepresentation of certain groups in the combined dataset, which could skew observed associations. Our descriptive analysis of the combined dataset presented in Table 1 shows that there is no meaningful overrepresentation of any demographic group beyond what is already present in the separate year datasets. shows that there is no significant overrepresentation of any demographic group beyond what is seen in the separate datasets. The demographic distributions in the combined dataset are largely consistent with those from the separate 2018 and 2019 datasets, with the exception of a slightly higher proportion of Black non-Hispanic respondents in 2019 (14% vs. 5% in 2018, resulting in 9% in the combined dataset). It is worth noting that sexual minority individuals of color and those from lower socioeconomic backgrounds are generally underrepresented in AMIS, which is a limitation inherent to both datasets. However, merging the datasets does not exacerbate this issue. Therefore, combining the datasets did not introduce any new overrepresentation of any demographic group or compromise demographic balance beyond distributions already present in the individual datasets.

Regarding potential variations in the stigma construct between the two years, we were mindful of this possible source of bias and conducted a three-step assessment to examine any inconsistencies in the measured stigma construct across the cohorts. Our analyses (previously detailed in the Appendix) showed that the stigma measures did not differ significantly between the 2018 and 2019 survey cohorts, indicating that the stigma measured in the combined dataset accurately reflected stigma across both years. With consistent stigma measures and other covariates across both years, we had no reason to believe that the associations between these variables would differ significantly across the separate year cohorts. Furthermore, there were no notable external events during this period that could have significantly altered stigma experiences for survey respondents.

We would also like to highlight previous studies, such as the Goodreau paper (combined 2017 and 2018 AMIS datasets) and the 2023 study by Ivey et al. (combined 2017 through 2020 AMIS datasets, <https://www.ncbi.nlm.nih.gov/pmc/articles/PMC10167950/>) which similarly used combined AMIS datasets. We strongly believe that merging the datasets offers several key advantages for our analysis. The larger dataset enhanced the detection of relatively modest but statistically significant effects, provided more precise estimates of these effects, and increased the overall robustness of our study findings—benefits that would be lost if only one dataset were used. These benefits are particularly important for our work that explored the interactions between sexual stigma and economic hardship involving eight groups, as the larger sample size ensured we had sufficient statistical power to examine these complex relationships.

However, we acknowledge that improvements could be made in how we presented points related to this issue. In response, we have made edits to the manuscript to ensure greater clarity. For example, we have added text in the Data source and study sample subsection (Methods) to highlight the small fraction of potentially repeat participants across years, addressing concerns about overlap and its potential impact on the analysis.

*Data from the 2018 and 2019 cycles of the American Men’s Internet Survey (AMIS), an annual cross-sectional behavioral health survey for GBM, was used.[37] AMIS annually reaches over 10,000 MSM who are recruited through banner advertisements on social media, geospatial networking apps, email blasts and various gay interest websites. Detailed information on AMIS eligibility, recruitment and enrollment processes have been previously described.[38,39] Data collection for AMIS 2018 occurred between September and December, while data for AMIS 2019 were gathered from August through December. Although AMIS is primarily cross-sectional, a small fraction (≤ 10%) of participants may have participated in previous survey years.[40]. [Manuscript clean copy, Methods section, Lines 66 – 74]*

We have also revised our discussion of the limitations associated with the data used in our analysis. The updated text now more clearly emphasizes the underrepresentation of racial minority groups and socioeconomically disadvantaged individuals in the AMIS data. This section of the manuscript now reads:

*This study had several limitations. While we used a diverse sample of GBM, it may not fully represent specific GBM demographics, particularly racial minorities, socioeconomically disadvantaged individuals, and HIV-status unaware GBM, affecting generalizability of the study findings. [(Manuscript clean copy, Discussion section, Lines 338 – 341)*

Additionally, we have revised the text in the Methods section to better highlight the assessment of stigma measurement invariance that was conducted to check for consistency in stigma measurement across the survey years. That portion now reads:

*To assess measurement consistency across the two survey years, a three-step process was applied: first, the optimal number of best-fitting latent classes was evaluated for invariance; second, class-specific conditional response probabilities were assessed for consistency; and third, class prevalence was examined.[57,58] Differences in the optimal number of classes or degradation of model fit in fully or partially constrained models indicated a lack of consistency (see Appendix). [Manuscript clean copy, Methods section, Lines 144 – 149]*

Text was also added in the results section elaborating on the findings of the stigma measurement invariance assessment.

*Stigma measurement invariance assessment showed consistent number of classes, conditional response probabilities, and class prevalences across both survey year data, confirming the stability of latent stigma measurement across the two cycles (Supplemental Table S3). [Manuscript clean copy, Results, Line 204 – 206].*

3. **Comment:** Additionally, I have questions regarding the use of multinomial logistic regression in the analysis. It is unclear whether dummy variables were used for the categorical variables, which is the recommended approach. Using dummy variables ensures that the categories are appropriately represented and compared in the regression models. Clarifying this aspect of the methodology would enhance the robustness of the analysis.

**Response:** Thank you for your comment. The multinomial logistic regression analysis was conducted appropriately in Mplus, where dummy variables were created for the categorical outcome levels. The minimal stigma group was designated as the reference level.

4. **Comment:** Moreover, the authors state: "Membership in diverse-SBS and FGSP-SBS classes were associated with younger age, past-year homelessness, poverty, and residence in the US South or Midwest" (page 9, line 182). However, based on the reported data, this is only true when compared to the reference class (which should be indicated in the table). Specifying the reference class used in the comparisons is crucial for accurately interpreting these associations.

**Response:** We thank the reviewer for highlighting this oversight. We have clarified that these associations are made in comparison to the reference group. Also to enhance sensitivity in our language, we have revised the manuscript to adopt a more person-centered approach. References to "membership" have been changed to emphasize individuals and their experiences, using phrases like "individuals in the diverse sexual stigma class" or "individuals identified as experiencing predominantly family or general sexual stigma experiences."

Specifically, the section referred to in the comment has been revised to read:

*“Participants identified as experiencing diverse stigma or predominantly family and general social stigma were more likely to be younger, have faced homelessness in the past-year, live in poverty, and reside in the US South or Midwest compared to those in the minimal stigma category (Table 2)”. [Manuscript clean copy, Results, Lines 210-212]*

5. **Comment:** Finally, the discussion should be revised based on the results obtained after the requested changes. This will ensure that the interpretation and implications of the findings are aligned with the revised analysis, providing a more accurate and robust conclusion.

**Response:** Thank you for your feedback. We have revised all relevant sections of the manuscript to address the issues raised. These revisions ensure that the discussion accurately reflects the results obtained, enhancing the interpretation and implications of our findings and leading to a more accurate and robust conclusion.

6. **Comment:** Minor Issues – Page 1, lines 9, 12, and 16: check citation format; Page 9, line 177: the title of a figure is inserted; Ensure that all software used for the analyses is properly cited and referenced.

**Response:** We thank the reviewer for these observations. We have corrected the citation format errors and revised the citations for Mplus and R. Regarding the figure titles, we retained them in accordance with journal guidelines, which state that "figure captions must be inserted in the text of the manuscript immediately following the paragraph in which the figure is first cited. Do not include captions as part of the figure files or submit them in a separate document."

**Reviewer 2 comments**

Thank you for the invitation to review this quantitative study examining the intersection of MSM stigma and poverty among MSM in the US. The analyses are straightforward, and the results have the potential to influence public health policies and interventions for this underserved group.

7. **Comment:** My main suggestion for the authors is to include more clarification around terminologies, methods, and analyses to make the manuscript more accessible to a wider audience. Many new terms are introduced in the latter part of the manuscript, and these should have been defined in the introduction section.

**Response:** Thank you for your valuable feedback. We have made concerted efforts to clarify terminology and reduce the use of abbreviations to enhance the accessibility of our manuscript. New terms introduced later in the text are now better defined, and we have provided explanatory context in the relevant sections to ensure smoother readability for a broader audience. For example, instead of using abbreviations for the four identified latent classes, we replaced them with more straightforward descriptions, allowing readers to follow along without needing to refer back to the results for clarification. Additionally, we have included clear explanations of methodologies that may be unfamiliar to some readers, such as latent class analysis. We defined it as follows:

*“LCA, a model-based clustering methodology, uses posterior probabilities of class assignment based on maximum likelihood estimation with robust standard errors to detect unobserved population heterogeneity from responses to observed indicators….”[Manuscript clean copy, Methods, Lines 137-139]*

We have retained detailed descriptions of methods in the Methods section to maintain clarity and ensure a coherent flow throughout the manuscript. To stay within the word limit, we have moved detailed descriptions of additional methodologies to the manuscript's Appendix and included references to them for readers who are interested in further details.

8. **Comment:** Line 3: Can clarification be provided on whether the authors are only referring to interpersonal stigma, or if they also include systemic, structural, and internal forms?

**Response:** Thank you for your comment. In the introductory section, we introduce sexual stigma as a multifaceted phenomenon. However, our study specifically examines individual-level manifestations of stigma, and we have added clarification to reflect this transition from describing stigma overall (a multifaceted phenomenon) to our specific focus on individual-level sexual stigma. The text now also explains the various forms of individual-level stigma identified in the literature.

*“Despite efforts to reduce discrimination [2–5], GBM continue to face stigma associated with sexual behavior, which profoundly affects mental health. This persistent challenge underscores the need for a deeper understanding of stigma as a multifaceted concept. Stigma involves distinguishing and labeling differences, associating those labels with negative stereotypes, segregating individuals into 'them' versus 'us’ categories, and ultimately subjecting labeled individuals to discrimination and status loss, resulting in unequal and adverse outcomes for affected populations.[6] Stigma operates at institutional, interpersonal, and individual levels. At the individual-level, sexual stigma manifests in various forms: as enacted or perceived stigma, where individuals experience direct or subtle discriminations based on their sexual identity; anticipated stigma, where individuals expect to face stigma in certain situations; and internalized stigma, where individuals accept a devalued identity as a part of his own value system and self-concept.[7–10]” [Manuscript clean copy, Background, Lines 3 – 14]*

We also recognize the importance of addressing other dimensions of sexual stigma beyond the individual level, including structural and systemic stigma. This acknowledgment is incorporated into the limitations section of the manuscript:

*“… Additionally, the focus on individual-level sexual stigma overlooks other dimensions of sexual stigma faced by GBM, including institutional, and community-level sexual stigma, as well as other stigma forms (mental health, HIV-related, etc.), along with their intersections with other systemic forces like racism that also affect mental health among GBM.” [Manuscript clean copy, Discussion, Lines 346 – 350]*

9. **Comment**: Page 1: While I appreciate the general description of stigma and its link to minority stress and psychological mediation, the concept of stigma should be further nuanced by addressing the specific injustices that MSM face.

**Response**: Thank you for your feedback. We have made efforts to highlight the specific injustices faced by MSM throughout the manuscript. For instance, we emphasize that the connections between stigma, minority stress, and psychological outcomes are not merely theoretical; they are supported by evidence that underscores the unique challenges and discriminations encountered by MSM:

*Empirical evidence supports these theoretical models, showing that sexual minorities face elevated rates of harassment, assaults, property violence, and other manifestations of sexual stigma.[13–16] Research also reveals a strong link between internalized homophobia and suicide outcomes, often compounded by heightened emotion dysregulation.[11,12,17–22] For instance, a longitudinal study of over 1,000 students found that adolescents with same-sex attraction exhibited greater emotional dysregulation, such as heightened rumination and reduced emotional awareness, compared to their heterosexual peers.[19] Similarly, another study showed that heightened rumination mediated the relationship between internalized stigma and depression.[18] Overall, the literature consistently shows that sexual minorities face an earlier onset and higher prevalence of mood disorders, substance use, and both suicidal ideation and attempts [23,24] compared to their heterosexual peers.[8,11,12,25].” [Manuscript clean copy, Background, Lines 25 – 35]*

10. **Comment:** Line 29: ad – typo?

**Response:** Yes, that was a typographical error, and it has been corrected. Thank you for pointing it out.

11. **Comment:** Line 32: Some justification is needed for prioritizing 'socioeconomic disparity' over other systemic forces like racism in this paper. I also recommend revising the title to more accurately reflect the paper's objective, as 'Intersecting Realities' is too broad for its scope.

**Response:** We appreciate the reviewer’s observation regarding the role of racism as a systemic force affecting mental health among GBM. While we acknowledge the significant influence of race on mental health disparities in U.S. populations, our study specifically focuses on the interplay of sexual stigma, poverty, and mental health. The impact of socioeconomic disparity on sexual stigma has been less explored in the literature compared to other systemic forces like racism, which can also shape and exacerbate these disparities. By prioritizing these factors, we aim to highlight their interconnectedness and address gaps in understanding how socioeconomic conditions influence stigma and mental health outcomes among GBM. We also acknowledge in the study limitations section that our focus may overlook other systemic forces that contribute to these disparities:

*“… Additionally, the focus on individual-level sexual stigma overlooks other dimensions of sexual stigma faced by GBM, including institutional, and community-level sexual stigma, as well as other stigma forms (mental health, HIV-related, etc.), along with their intersections with other systemic forces like racism that also affect mental health among GBM.” [Manuscript clean copy, Discussion, Lines 346 – 350]*

Race was included in our analyses as a confounding variable to control for its influence and ensure the validity of our findings. Although we recognize the importance of racial disparities, we chose not to explore them in depth to maintain focus on the unique contributions of sexual stigma and poverty. Racial data are available in Table S1 for reference and further research. Future studies could build on our findings by examining how these factors interact with race and ethnicity as markers of racism influencing mental health among GBM.

Additionally, we have revised the title to better reflect the core focus of the study: Exploring the Intersections of Sexual Stigma, Poverty, and Mental Health in HIV-Negative Gay, Bisexual, and Other Men Who Have Sex with Men in the United States.

12. **Comment:** Line 39: Can I recommend citing Carmen Logie's work on identifying sexual orientation (including identity, behavior, etc.) as a determinant of health, alongside other crucial factors such as socioeconomic status, to strengthen your case for examining these multiplicative determinants of health? [https://doi.org/10.2105/AJPH.2011.300599](https://nam11.safelinks.protection.outlook.com/?url=https%3A%2F%2Fdoi.org%2F10.2105%2FAJPH.2011.300599&data=05%7C02%7Cudodirim.onwubiko%40emory.edu%7C8132d76ec12849e89cc708dcd14e4202%7Ce004fb9cb0a4424fbcd0322606d5df38%7C0%7C0%7C638615381286184516%7CUnknown%7CTWFpbGZsb3d8eyJWIjoiMC4wLjAwMDAiLCJQIjoiV2luMzIiLCJBTiI6Ik1haWwiLCJXVCI6Mn0%3D%7C0%7C%7C%7C&sdata=UqkvmjgqsvpuCtmKDF4diQ32JR9roOkEtq0vOsOA9Gg%3D&reserved=0)

**Response:** Thank you for the suggestion. This has been incorporated.

13. **Comment:** Page 56: Add a sentence about the recruitment strategy.

**Response:** Thank you. This has been added.

14. **Comment:** Line 70: A sentence is needed regarding the validity and reliability of the K6 measure for this sample.

**Response:** Thank you. This has been added.

15. **Comment:** Line 78: A sentence explaining how these sexual stigma items were designed or chosen needs to be added.

**Response:** Thank you for your feedback. The stigma items used in our analysis were selected from a consistent set of survey questions measuring firsthand sexual stigma experiences among AMIS participants from 2017 to date. While other questions exploring sexual stigma have been added and removed in AMIS over time, we opted to only use those that have remained consistent over time to enhance comparability with findings from studies using similar measures. We have added the following text to clarify this point:

*“Fifteen AMIS survey items (Supplemental Table S0) were used to measure individual-level sexual stigma experiences across various contexts, all of which have been included in AMIS surveys since 2017.”[Manuscript clean copy, Methods, Lines 97-99]*

16. **Comment:** Line 85: A brief definition is needed in the introduction section about enact and anticipated stigma.

**Response:** Thank you for your comment. We have added a brief definition of enacted and anticipated stigma to the introduction. The updated text now reads:

*“At the individual-level, sexual stigma manifests in various forms: as enacted or perceived stigma, where individuals experience direct or subtle discriminations based on their sexual identity; anticipated stigma, where individuals expect to face stigma in certain situations; and internalized stigma, where individuals accept a devalued identity as a part of his own value system and self-concept.[7–10].” [Manuscript clean copy, Background, Lines 10 – 14]*

17. **Comment:** Line 88: It will be acceptable to refer to 'sexual orientation' here only if the introduction includes a definition that broadly encompasses sexual identity, behavior, preference, etc.

**Response:** Thank you. This has been changed to say “sexual behavior” instead.

18. **Comment:** Line 89: More clarification is needed about the categorization of stigma in different settings. Not quite sure what line 89 means. Can the language be simplified or more elaboration be added?

**Response:** Thank you for your feedback. We have clarified the ambiguous language in the line referred to by providing additional details. The revised text explains the categorization of stigma in different contexts. Furthermore, we have included the specific stigma survey items used in our analysis as Supplemental Table S0. This table allows readers to see the exact wording of the items measuring stigma experiences across various settings. We have also added the citations to provide more background on the stigma items used.

19. **Comment:** Page 6: Further clarification (in layman terms) is needed on how the categories are determined: diverse forms of stigma across multiple settings; primarily anticipated stigma in healthcare settings; predominantly enacted and perceived sexual stigma in family and general social settings; or minimal sexual stigma.

**Response:** Thank you for your insightful comment. We recognize that our description of the categories of sexual stigma in the PrEP outcomes regression analyses was not fully clear. To clarify, the stigma categories were determined using latent class analysis (LCA), as explained in our Methods section. In this process, we first converted the stigma-related AMIS items into binary indicators of lifetime stigma experiences (n = 13). We then used these indicators in statistical models to identify groups of individuals with similar stigma experiences based on their responses. The best fitting LCA model revealed four distinct sexual stigma groups in the study sample, each characterized by a unique pattern of stigma items response probabilities (see Supplemental Table S2 for these response probabilities). The labeling of each identified stigma class was based on these estimated response probabilities. For example, Class 2, which we labeled as “the Anticipated Healthcare Predominant Sexual Stigma Class”, was distinguished from other classes by the very high probabilities individual’s in this class had for endorsing two specific survey items: i) "Have you ever felt afraid to go to healthcare services because you worry someone may learn that you have sex with men" (response probability = 0.94), and ii) "Have you ever avoided going to healthcare services because you worry someone may learn that you have sex with men" (response probability = 0.84). The other three classes were similarly labeled according to their patterns of response probabilities across the 13 stigma indicators as estimated in the same LCA model.

To improve clarity around the stigma measure used, we have revised the footnote accompanying Table S2 to read:

*“…**Latent stigma classes: Class 1: labeled “the Diverse Sexual Stigma Class” – Characterized by high probabilities of endorsing various types of stigma experiences across different settings. Class 2: labeled “the Anticipated Healthcare Predominant Sexual Stigma Class” – Defined by a particularly high probability of endorsing anticipatory stigma in healthcare settings. Class 3: labeled “the Family and General Social Sexual Stigma Class” – Marked by moderate-to-high probabilities of endorsing stigma related to family and general social settings, but not in healthcare settings. Class 4: labeled “the Minimal Sexual Stigma Class” – marked by generally low probabilities of endorsing any of the surveyed sexual stigma experiences.”*

Additionally, we have updated the relevant portion of our results to provide a clearer description of the identified latent stigma classes. The revised text is as follows:

*“Examination of conditional response probabilities (Fig. 1B) estimated in the 4-class model revealed distinct patterns of stigma experiences, grouping GBM into: 1) those with diverse stigma experiences across multiple settings (group labeled as the “Diverse Sexual Stigma class”), 2) those with predominantly anticipated stigma in healthcare settings (labeled “the Anticipated Healthcare Predominant Sexual Stigma class”), 3) those with predominantly enacted and perceived sexual stigma experiences in family and general social settings (“the Family and General Social Sexual Stigma class”), and 4) those with generally minimal stigma experiences (“the Minimal Sexual Stigma class”).” [Manuscript clean copy, Results, Lines 190 – 197]*

20. **Comment**: Line 227: Definitions are needed for 'multiplicative' and 'additive'.

**Response:** Thank you for your insightful comment regarding the need for definitions of "multiplicative" and "additive." We appreciate your suggestion and have included references to relevant literature as well as documentation in the Appendix that provides a more detailed description of these well-established concepts than we could fit within the manuscript's word limit. Additionally, we aimed to clarify their implications in the results section by stating:

*"However, a super-additive (or greater-than additive) interaction was observed for all associations, indicating that the co-occurrence of patterns of sexual stigma experiences exceeding minimal levels and poverty was associated with excess prevalence of all three mental health outcomes beyond the expected sum of prevalences when either condition occurred alone." [Manuscript clean copy, Results, Lines 264 – 268]]*
